# Understanding Decision Making in Airway Management Using a Cognitive Task Analysis Approach: A Case Study in the Emergency Department Resuscitation Area

**DOI:** 10.7759/cureus.98007

**Published:** 2025-11-28

**Authors:** Daniella Cooper

**Affiliations:** 1 Department of Medical Education, The Academy, Great Western Hospital, Swindon, GBR

**Keywords:** cognitive task analysis, decision making, emergency airway management, emergency department, human factors

## Abstract

Background: Healthcare advances are rapid, as are the changes in human factors approaches to healthcare, such as designing safer workplaces and improving communication and teamwork. It is necessary for work to be done to improve the congruency of these for patient safety and the efficiency of systems. Emergency departments (EDs) are rapidly changing environments, so it is difficult for staff to adapt to them.

Objectives: This project explores critical themes where applying a human factors approach to decision-making during emergency airway management in the ED resuscitation area (ED resus) can enhance patient safety, optimise team performance, and improve overall system efficiency.

Methods: A homogenous sample of 25 frontline staff from operating theatres and ED at Queen's Medical Centre, Nottingham, England, were interviewed using a cognitive task analysis (CTA) technique to determine what affects decision-making regarding emergency airway management in resus. Transcripts were coded using NVivo 12 software (QSR International, Burlington, MA, USA) and analysed using thematic analysis techniques to identify key themes for areas of improvement.

Results: The themes that emerged from analysis were split into system components and decision-making factors about emergency airway management. System components consisted of the equipment present and the training that staff receive. Decision-making factors included cognitive elements, planning, policies and guidelines, and teamwork. The emergent themes reflected the use of healthcare systems frameworks in terms of the human factors approach.

Conclusions: This study exhibits the use of human factors and the CTA method to discover specific recommendations for the improvement of patient safety and systems efficiency regarding emergency airway management in ED resus. These recommendations need to first be reviewed by a high-level systems approach before being implemented in the clinical setting.

## Introduction

The aims of this project were to explore critical themes where applying a human factors approach to decision-making during emergency airway management in the emergency department (ED) resuscitation area (resus) can enhance patient safety, optimise team performance, and improve overall system efficiency. Human factors is a scientific approach to optimising the relationship between the environment, equipment, and staff to improve performance, safety, and patient care. Cognitive task analysis (CTA) is a method that aims to understand the mental processes involved in completing a task, especially aimed at understanding tasks that require a lot of cognitive activity. A CTA approach was used in this project to determine the depth of decision-making and human factors that can affect it.

Healthcare advances have been rapid, especially in technological fields; however, they are still somewhat behind in human factors approaches [1]. The basis of human factors is to understand a system and optimise it to make it easier to do the right thing and harder to do the wrong thing [2]. Implementing human factors approaches will improve patient safety and improve the efficiency of the system by seeking to eradicate the underlying problems, resulting in longer-term learning and lasting change [1]. This is especially true within clinical areas such as the ED, where some aspects of care rely on decision-making to be made in fast-moving, dynamic, and unpredictable situations. Errors often result from cognitive overstimulation in stress, leading to impaired decision-making, fixation, omission, and failure to act [3]. Teamwork has often been commented upon as affecting the decision-making process. This could be due to different staff groups interacting without much practice, which could be counteracted by joint training, such as interprofessional learning simulations [4]. It is known that a team that communicates well, defines the roles of its members, and is aware of their limitations will provide safe patient care [5].

The definition of a difficult airway is one that cannot be secured in a reasonable number of attempts using usual methods [6]. Difficult intubation often arises unexpectedly, the incidence of which is unknown; however, it is thought to be around 1%-3% [7]. There is a need for more attention on human factors for emergency airway management to improve outcomes and to improve the steps: limiting procedural attempts, recognising failure promptly, and transitioning to the next stage of the algorithm [8]. Incidents are rarely caused by one wrong decision or action; they are generally caused by interactions between people, tasks, technology, and working conditions. There are many guidelines that exist for emergency airway management; however, many of them don’t specify at which stage they should be used (at preparation or implementation) [3]. This renders them ineffective and can make them disruptive to the team, the opposite of what they are supposed to achieve. The concept of an ‘airway time-out’ is emerging and is commonly included in guidelines now to prevent fixation and to ensure all team members are aware of the situation [9]. Regular high-fidelity simulation training of airway protocols will help to ingrain airway skills in daily practice [10].

Previous studies into human factors affecting airway management include the 4^th^ National Audit Project (NAP4), carried out by the Royal College of Anaesthetists and Difficult Airway Society. It highlighted that in the ED, airway management was poor in 46% of cases, with procedures performed more often out of hours by doctors with less anaesthetic experience because consultants were often not present, resulting in permanent harm [4]. NAP4 identified that there were human factor issues involved in 40% of the cases. In a 2013 study that interviewed some clinicians from NAP4, common issues identified were situational awareness deficits leading to ‘failure to anticipate’ and wrong decision-making [11]. Another study in 2018, in response to NAP4, investigated airway management of critically ill patients in all hospital locations; they identified problems in several areas, including equipment, training, and teamwork [8]. It was recommended that a standardised trolley be implemented to overcome these issues. Teamwork was made difficult due to inconsistent members from multiple professions being involved. This can be improved by using structured algorithms, team briefs, checklists, handovers, leaders and followers, and potentially joint training [8].

## Materials and methods

Qualitative data collection was used in this project as it allows for a more detailed analysis of the decision-making processes involved [12]. It enables us to focus on personal experiences to provide deeper meaning and context, rather than gather information that can be generalised [13]. In this project, it was necessary to use purposive sampling, a technique where researchers intentionally select participants based on their specific knowledge, characteristics, or experiences, to recruit participants who were relevant rather than randomised [14]. The sample needed to be both adequate in size and appropriate in experience levels [15], and the related interests and specialities of the participants needed to be considered to ensure a homogenous sample was achieved. A homogenous sample of 25 frontline staff from operating theatres and the ED at Queen's Medical Centre, Nottingham, England, was interviewed. A detailed breakdown is presented in Table 1.

**Table 1:** Description of interviewees showing how many of each level of training or number of years qualified. CT: core training (e.g., CT3 is three years into core training); ST: speciality training (e.g., ST6 is six years into speciality training); ODP: operating department practitioner; ED: emergency department

Staff group	Level/ years qualified	Number of interviewees
Anaesthetists	CT3	1
	ST6	3
	ST7	4
ODPs	3 years	1
	5 years	3
	7 years	1
	8 years	1
	10 years	1
	15 years	1
ED doctors	ST3	1
	ST5	1
	Career middle grade for 10 years	1
	Consultant	2
ED nurses	1 year	1
	2 years	3

Interviews were used in this project as an adaptable way to follow up on ideas and use probing questions to investigate the motives and feelings of the participants (Appendix A contains the interview schedule). The interviews lasted between 25 minutes and one hour and were audio recorded to assist with data analysis. CTA was used in this project, exploring tasks that rely on cognitive aspects of expertise, for example, problem-solving and decision-making. CTA is a ‘bottom-up’ approach, as it starts with identifying a cognitive challenge but with no clear design goal at the end [16]. Consent was gained from all participants by means of a consent form. A participant information sheet was supplied for information before the consent form was completed. Ethical approval for this project was gained from the Faculty of Medicine and Health Sciences Research Ethics Committee (approval number: 409-1910).

The transcripts from the interviews were coded using NVivo 12 (QSR International, Burlington, MA, USA) and analysed using thematic analysis, which captures the importance of data and identifies patterned responses. The analysis was completed alongside the interviews to allow spiral analysis, taking concepts from previous interviews and incorporating them into future interviews to build data in the ‘lacking’ areas [15]. Themes were weighted according to their prevalence among participants and the richness of detail provided; those mentioned more frequently were considered more significant and thus received greater attention in the analysis. An important limitation was the potential issues with reliability and validity in this project; for example, would participants give the same information if interviewed at a different time, and would different analysts take the same codes from raw data [17]? In terms of the actual data, memory degradation leads to concerns with data reliability [18].

## Results

Themes from the analysis were separated into systems and decision-making. Table 2 describes both the number of transcripts each node was coded from (files) and the number of times each node was coded for in total (references). Table 3 describes the number of times each node was coded for by each staff group.

**Table 2:** Description of nodes and number of times they have been coded Nodes: themes; Files: number of transcripts coded from; References: number of times coded in total

	Node (sub-node)	Files	References
Systems	Equipment	7	8
	(Positive)	23	72
	(Negative)	23	77
	(Improvement)	20	37
	(Medication)	23	37
	Training	8	14
	(Current)	24	47
	(Improvement)	25	52
Decision-making	Cognitive Elements	17	28
	(Distraction)	9	14
	(Night shift)	22	35
	(Stress)	7	12
	(Time constraint)	4	8
	(Tiredness)	17	22
	Planning	24	83
	(Back-up plan)	24	35
	(Goals)	21	25
	Policies and Guidelines	21	50
	Teamwork	7	9
	(Help)	24	115
	(Hinder)	22	49
	(Improvement)	7	11
	(Roles)	25	69
	(Staffing)	14	23

**Table 3:** Description of nodes and number of times they have been coded in total and by each staff group ODPs: operating department practitioners; ED: emergency department

	Node (sub-node)	Total	Anaesthetists (/8)	ODPs (/8)	ED Doctors (/5)	ED Nurses (/4)
Systems	Equipment	7	0	2	4	1
	(Positive)	23	7	7	5	4
	(Negative)	23	8	7	5	3
	(Improvement)	20	5	7	4	4
	(Medication)	23	6	8	5	4
	Training	8	2	2	4	0
	(Current)	24	7	8	5	4
	(Improvement)	25	8	8	5	4
Decision-making	Cognitive elements	17	3	7	4	3
	(Distraction)	9	3	4	2	0
	(Night shift)	22	7	7	4	4
	(Stress)	7	6	0	1	0
	(Time constraint)	4	2	1	1	0
	(Tiredness)	17	7	4	4	2
	Planning	24	8	7	5	4
	(Back-up plan)	24	8	7	5	4
	(Goals)	21	7	8	3	3
	Policies and guidelines	21	6	7	4	4
	Teamwork	7	0	3	1	3
	(Help)	24	7	8	5	4
	(Hinder)	22	8	7	3	4
	(Improvement)	7	4	2	1	0
	(Roles)	25	8	8	5	4
	(Staffing)	14	3	5	4	2

Systems

Equipment

Analysing equipment, most interviewees (n=22) reported that when the bays are well stocked, they have all the equipment they need for any airway situation. Three interviewees mentioned how helpful standardised trolleys are in each bay, so they know where to get the equipment from. The intubation trays were highlighted by three operating department practitioners (ODPs) and one ED doctor as being extremely useful to aid in quick setup for emergency situations. It was mentioned that the difficult airway trolley mimics the one in theatres, helping to decrease cognitive load in an emergency. Negative points mentioned included six anaesthetists declaring how difficult it is when equipment is not stocked up properly or is hard to find. It was suggested that the airway trolleys be sealed or made so that the drawers cannot be removed to help with the stocking issues.

Training

When asked about current training, seven interviewees mentioned teaching classes, simulations, and exams, and four stated that simulation is useful. Anaesthetist 1 recognised that “people who learn how to just do things like that from a book are often not very good at doing it”. Also suggested was elective airway management, helping to build skills, and the benefit of regular difficult airway workshops. In terms of suggested improvements, six interviewees recommended that joint training with theatre and ED staff would be beneficial and would help to understand each other’s roles more. Two interviewees suggested in situ training in resus itself, which would also help to improve team performance. Five interviewees advocated practising non-technical skills in these sessions too, such as leadership and followership skills, which ODP 8 highlights the importance of by indicating, “Often when I do the Datix and incidents and things, often what’s gone wrong is not often clinical; it’s often human factors that have led to it, and one of the big things is communication”.

Decision-making 

Cognitive Elements

**Figure 1:**
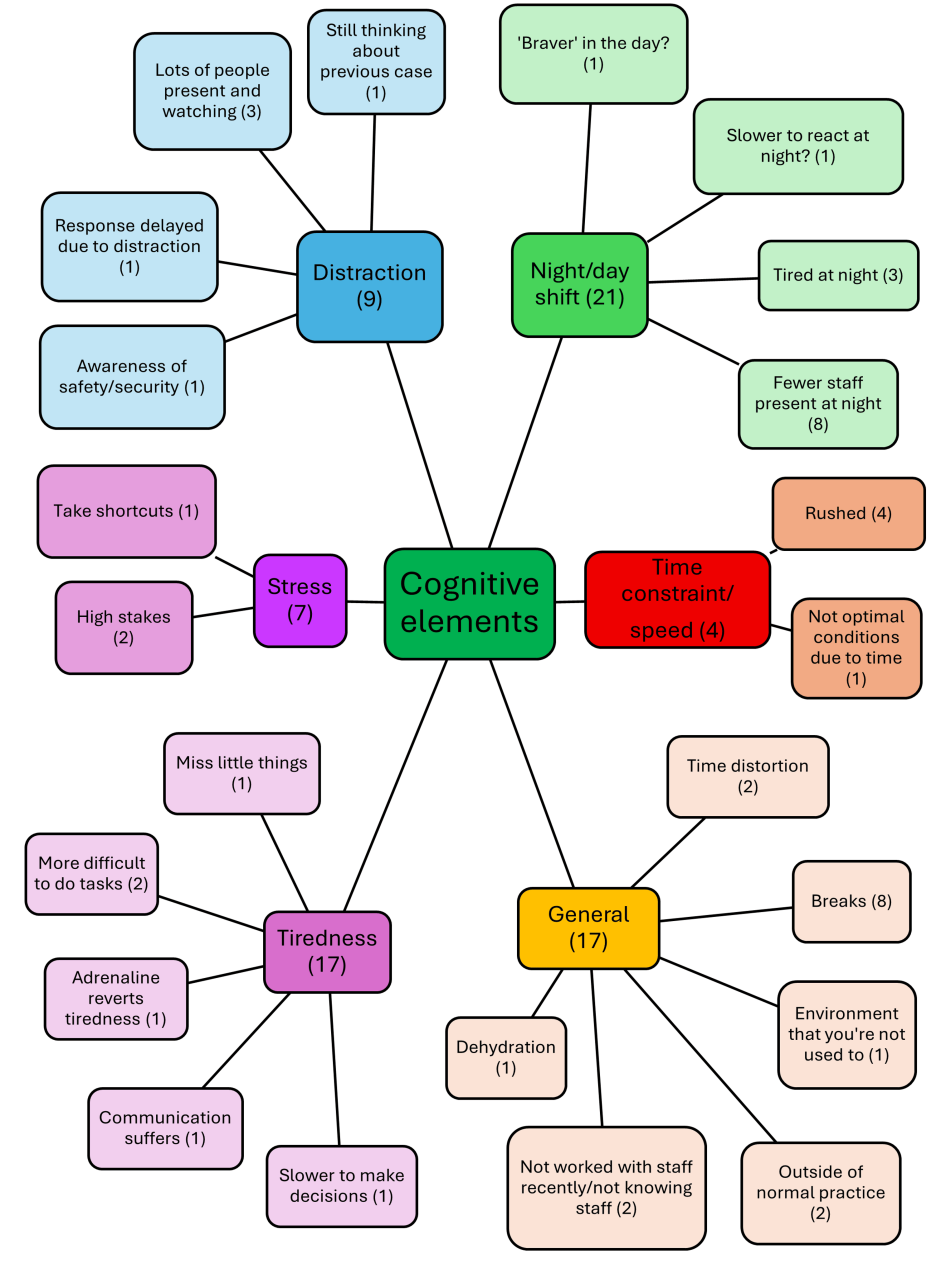
Description of cognitive elements identified by interviewees that affect decision-making regarding emergency airway management in ED resuscitation, including the number of interviewees who mentioned each ED: emergency department This figure has been created by the author.

Cognitive elements are things that affect your cognition whilst you are making decisions. ODP 2 stated, “I wouldn’t like to think that it would affect the quality of your decisions or the speed, but it definitely plays a part in the human factors, things getting missed off”. Night shifts make decision-making trickier. ED Doctor 5 mentioned how “you can often feel more fatigued in your ability to do procedural tasks from memory”. It was discussed by ODP 1 that “we can probably take a few more risks in the day that we wouldn’t at night because the safety net is there,” as there are fewer staff present at night, and getting senior support is slower. Table 3 shows that tiredness was mentioned by almost all anaesthetists and ED doctors, but only by half of ODPs and ED nurses. Breaks are important for your brain to rest and for you to rehydrate and nourish yourself. ODP 5 explains, “If you’re really hungry, or if you’re really dehydrated, you’re not going to be thinking quite as clearly”. There are more cognitive elements that also affect decision-making, illustrated in Figure 1.

Planning

**Figure 2:**
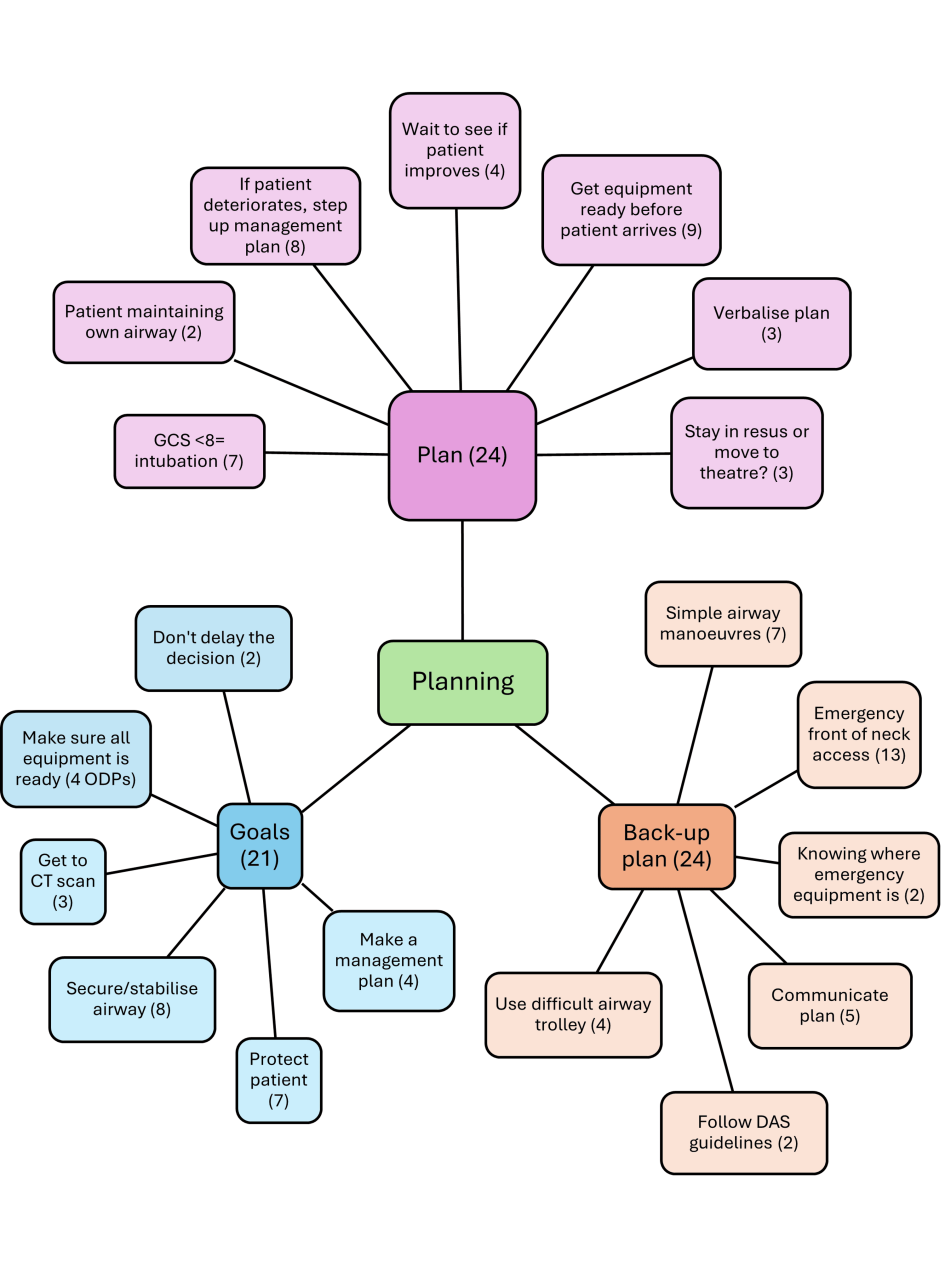
Description of the components of plans, backup plans, and goals regarding emergency airway management in ED resuscitation and the number of interviewees who mentioned them ED: emergency department; DAS: Difficult Airway Society This figure has been created by the author.

Planning is very important in the decision-making process. The plan needs to lead to specific goals, and there also needs to be a backup plan in place to ensure safe care. Figure 2 shows the main themes that occurred, along with how many interviewees mentioned them. ODP 2 highlights the need for a plan - “I feel like we should always have a well-laid-out plan so that the entire team is on the same wavelength of what should happen for each step”. In terms of a backup plan, Anaesthetist 3 stated, “I didn’t announce a backup plan. So, that would have probably been useful to have done”.

Policies and Guidelines

When asked if they had used any policies and guidelines to aid their decision-making, Anaesthetist 3 stated, “the airway point of view is relatively straightforward, as there are guidelines and protocols that suggest this is what you need to do in this situation”, and ODP 4 declared, “The algorithms are in place so I don’t have to think about what I should be doing next”. Eight interviewees mentioned the Adult Life Support (ALS) guidelines, nine mentioned the Difficult Airway Society (DAS) guidelines, six mentioned that if Glasgow Coma Scale (GCS) score is less than 8, then intubation is recommended, three suggested the use of checklists, and five stated they had not read anything at the time to help with their decision-making, supported by a quote from ED Nurse 1: “there probably is guidelines available for that, but again, I just knew it because I’ve worked in that situation a lot now”. Of those who mentioned DAS guidelines, two were anaesthetists, one was an ED doctor, and six were ODPs. ALS was split into one anaesthetist, three ED doctors, two ED nurses, and two ODPs.

Teamwork

**Figure 3:**
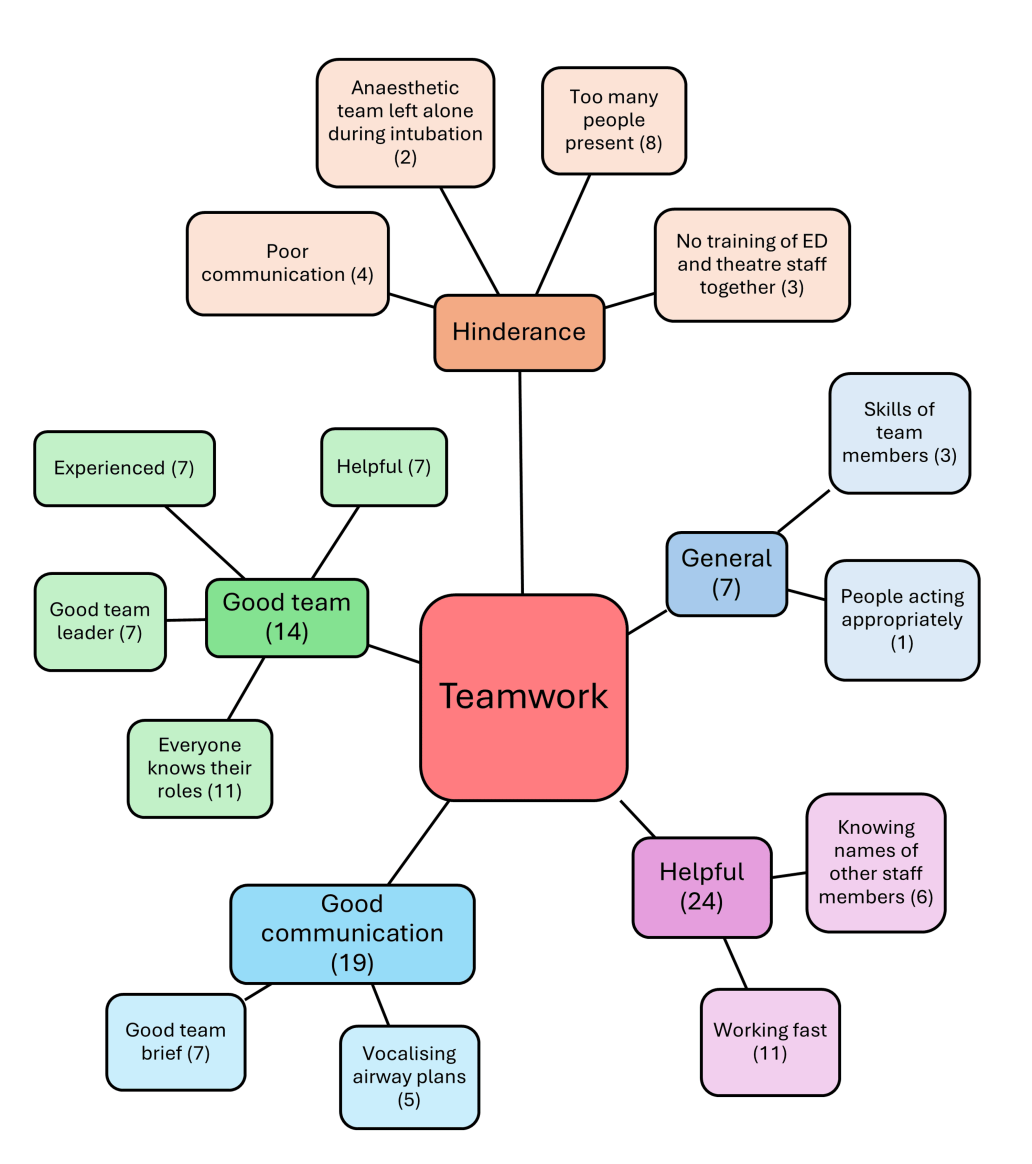
Description of the components of teamwork regarding emergency airway management in ED resuscitation, and the number of interviewees who mentioned them ED: emergency department This figure has been created by the author.

Teamwork is essential in healthcare, especially in the emergency environment. Having a good team that is helpful and experienced is indispensable, as well as a good team leader, and when everybody knows their roles. Most interviewees (n=23) mentioned that clear roles were identified, and Anaesthetist 6 mentioned, “I think in that situation, I think we’d probably allocate roles we felt were necessary. I think you can end up having too many people doing too many things”. Having too many people around was identified by eight interviewees as being a hindrance. Eleven interviewees recognised that good teamwork is identified by working at a good speed, and ED Doctor 4 proposed the need to “recognise that it’s more stressful and use things like checklists and give a bit of time to work together to make sure we all understand it and recognise that this is unsafe”. This comes with good communication, which was highlighted by a significant number of interviewees (n=19) as being helpful; this includes vocalising airway plans, having a good team brief, and knowing the names of other staff members. Figure 3 describes some more teamwork-related topics that arose.

## Discussion

Systems

Equipment

One theme that emerged through analysis was the importance of having the correct equipment available, such as trolleys being well stocked. This indicates that more importance needs to be placed on restocking. It would also be helpful if the trolleys were labelled better, clutter removed, and equipment made more visually accessible, so staff know what is available. Having equipment in view can help remind staff that there are alternative options to try when difficulties occur [12]. The intubation trays also aid in this and allow for quick preparation during emergencies. It was noted that the difficult airway trolley in resus mimics the one in theatres, helping to decrease cognitive load in emergencies, which will decrease error and confusion [4, 8, 12]. 

Training

When asked about training, Anaesthetist 5 said, “We are lucky compared with other hospitals for being well trained and managing airways,” whereas ODP 3 said, “Training is little and not very often, in all honesty”. There is a discrepancy here that potentially needs reviewing. Training could be improved by conducting more simulations and apprentice-style teaching with more experiential learning [8]. A quote from ED Doctor 5 states, “I don’t think that our trainees get the same experience that I and some of my colleagues at my level get; it can’t be the same. You can’t be as happy dealing with a critical patient in that situation”. However, this will only work if staff are targeted more systematically so no one is missed. Joint training between theatre and ED staff, such as simulations, will help both teams to understand each other’s roles better and improve teamwork skills [4].

Decision-making

*Cognitive Elements* 

In terms of cognitive elements, the findings suggest that staff are subject to an increased cognitive load when exposed to an environment that they are not used to [8]. Cognitive load is the amount of effort and information-processing demands placed on your working memory at any one time. If this is overloaded, the brain exceeds its capacity to process information, leading to errors and decreased quality of care. Stress was described by interviewees as arising from high-stakes situations and leading to shortcuts being taken. Errors often result from cognitive overstimulation in stress, which can lead to impaired decision-making [3], and the rise in cortisol due to stress leads to changes in cognition and behaviour and causes loss of accurate recall of information [19]. ED Doctor 3 noted this by stating, “Your ability to stay calm in a crisis is more important than the ability to make a decision,” as if your stress levels rise, it will be even more difficult to make that decision. Night shifts result in tiredness; interviewees mentioned that this caused slower reactions, more difficulty in performing tasks, and communication suffering. Fatigue, shift work, and sleep deprivation can lead to errors, possibly due to these factors [1].

Planning

When comparing the planning components of the transcripts to the DAS guidelines, many of the steps correspond, such as if the patient deteriorates, step up management plan, back-up plans including simple airway manoeuvres, emergency front of neck access, and the difficult airway trolley. However, it was also mentioned that equipment is prepared in advance, and waiting to see if the patient improves, the judgment of this comes from experience and confidence, and taking equipment to scans in case it is needed. These extra decisions are due to the nature of the airway intervention being an emergency situation rather than routine in theatres.

Policies and Guidelines

Guidelines are suggested by the findings to help make the decision-making process more straightforward and ensure everyone is on the same page so that effective teamwork can happen, highlighted by ODP 2’s quote “I don’t think I’ve ever had that disagreement because they’re aware of the algorithm, I’m aware of the algorithm, we’re all singing from the same hymn sheet”. However, it was mentioned that if there isn’t much time, especially in an emergency situation, guidelines can slip away. An ED doctor mentioned that they are awaiting a rapid sequence induction checklist to help anaesthetists when coming down from theatres to resus, as the change in environment can be a hindrance to decision-making and can increase the cognitive load.

Teamwork

When you have a good team, decision-making is easier. A good team is made when staff are helpful, experienced, and everyone knows their role, which is helped by the identification stickers that are used during trauma calls. Good communication is key and is aided when the names of staff members are known, when a team brief occurs, and when plans are vocalised. A good team leader is essential and can help to maintain situational awareness [20]. A major hindrance is when there are too many people present, or you don’t know the staff, which can be counteracted by joint training [4]. The data provide evidence that there are fewer staff on night shifts, which affects how supported staff are and the ability to make more complex decisions, as evidenced by a quote from ODP 1: “We can probably take a few more risks in the day that we wouldn’t at night because the safety net is there”. ED Doctor 4 stated the need to “recognise that it’s more stressful and use things like checklists and give a bit of time to work together to make sure we all understand it and recognise that this is unsafe”. Structured algorithms, team briefs, checklists, and handovers can all help to improve teamwork and patient safety [8].

Limitations

There were limitations in this project on the sample size and how many interviews could be carried out due to time constraints. Due to this, it is important to state that a small representation of certain themes does not mean that they are not important, and there is a potential that certain themes may have been missed. Not all themes that arose during interviews have been analysed and discussed due to the vast amounts of data. To decide which themes to analyse, certain factors were considered, including how passionate interviewees were about certain topics and how much information was discovered. For example, experience levels and situational awareness were not explored in depth due to time constraints, so this could be an area for further work. There could be reflexivity bias as only one researcher carried out the interviews and analysis, so there is a potential for unconscious subjective influence of the researcher's beliefs, experiences, and preconceptions, which could impact the objectivity and validity of findings. Preferably, the coding should be validated by another researcher as well to improve inter-rater reliability. As the study was conducted at a single centre, its findings may have limited generalisability and may not be fully applicable to other settings. To improve the transferability of results, future research should include multi-centre studies across diverse environments and incorporate larger, more varied participant samples.

## Conclusions

This study applies human factors principles alongside the CTA method to identify actionable recommendations aimed at enhancing patient safety and improving system efficiency in emergency airway management within the ED resus. As this is an in-depth, single-centre qualitative investigation, the proposed recommendations should be evaluated through a broader systems-level review prior to implementation in clinical practice. The analysis indicates that optimal performance occurs when processes function as intended, resulting in staff satisfaction and successful task execution. Sustaining these conditions requires structured interventions, such as ensuring equipment is consistently stocked and clearly accessible, adopting experiential and interprofessional training approaches, and appointing a team leader to manage staffing, clarify roles, and maintain situational awareness. These recommendations represent only a subset of potential improvements. Future research should incorporate multiple transcript reviewers to mitigate reflexivity bias and extend the scope of analysis to capture the complexity of the clinical environment, decision-making processes, and influencing factors.

## Appendices

Appendix A 

**Table 4:** Interview schedule used including sections for specific staff ED: emergency department; CT: core training; ST: specialty training; QMC: Queen's Medical Centre; ODPs: operating department practitioners

Theme	Questions
General	So first we will run through a few background questions, those shouldn’t take long and then I will ask a few questions about the ED Resus area, before I ask you to focus on one example of decision making in airway management and how that was experienced.
Background	What is your clinical role?
	What stage of training are you in (CT/ST level)/ how many years have you been qualified?
	How long have you been at QMC for this attachment/ in your current role?
	Have you had any previous attachments here?
General	Ok, so now I’d like you to think specifically about your experience of working in the ED resuscitation area. We are particularly interested in finding out about how people experience work and decision-making regarding acute airway management in that working environment.
ED Resuscitation Area	What patients/ cases are you expected to see in ED?
	When you see them, what specific aspects of care are you involved with?
	Thinking about ED, is there anything you particularly like about the ED resus bay?
	Are there any aspects that help or hinder you when you go to see a patient?
For theatre staff only	For the purpose of this study and the questions that will be asked, we would like you to think about a case that you have experienced or might realistically be expected to help with in the ED resus area. In particular we would like you to think about a patient who has arrived in ED resus with a significant degree of airway compromise and which the ED staff have asked you to manage as part of the overall patient assessment. This could be a patient with major trauma, or altered conscious status, or for some other reason.
For ED staff only	For the purpose of this study and the questions that will be asked, we would like you to think about a case that you have experienced or might realistically be expected to help with in the ED resus area. In particular we would like you to think about a patient who has arrived in ED resus with a significant degree of airway compromise for which you have called for anaesthetic assistance, but that team has not yet arrived.
General	I would like you to think about something quite recent, and we are going to explore this example in depth, so please pick an example that you are happy talking about. We are going to think about the decisions you had to make. (ODPs- Specifically, the decisions you made without being asked by the anaesthetist, ED staff- whilst you were waiting for the anaesthetic team to arrive) Please can you give me a brief overview of your decision and we will map it out into a timeline using post-it notes. And within this timeline please can you pick a decision point, one that may be affected by the context and other things around you. Now we’ll think about this decision in more detail.
Decision	What decision did you make and why?
	How did you know it was the right thing to do?
	What were your specific goals at this time?
	Are there any reasonable alternatives, at the time or in hindsight?
	Are there any common potential errors at this point?
Teamwork	Thinking about teamwork, who was involved in this scenario? (was your ODP there, what if they weren’t)
	Were the roles of the team members clear?
	What went well/not so well?
	Did anyone question your decision?
	What happens if you disagree with the anaesthetist’s plan for airway management?)
Cognitive Elements	Can you think of any difficult cognitive elements associated with this situation? (for example, were you tired- from day or night shifts, was there much pressure on you, had you had your break)
Location and environment	Thinking about the location and environment of the ED Resus Bay, are there any things that help or hinder your decision making? (e.g. enough space, things in the way?)
Equipment	Now thinking about equipment, were there any factors that affected you regarding that? (e.g. was it accessible, what you are used to using)
	Are there any ways you think equipment could be improved?
	Whose responsibility is it for maintaining stock in the bays?
	What do you think about access to medications?
Training and experience	Let’s think about training and experience now, what kind of experience/training would be needed to make this kind of decision? What training have you had?
	Were you reminded of a previous experience?
	Was this a routine case or were there any unusual elements to it?
	Could you make the decision with less experience?
	Is there any training you think would be useful to help you or others in the future?
Cues and strategies	Thinking again about the environment you were in, where there any cues or strategies that you remember, such as seeing or hearing anything?
Information	Was there any explicit information that you used to make this decision?
	How did you get this information and who from?
	Did you use any guidelines or decision aids?
Planning/ situational awareness	Did you imagine the possible consequences of the decision you made?
	Did you imagine how events might unfold and was there a back-up plan?
	After making the decision, when was the next opportunity you had to review it?
	How long did it take you to make the decision?
	Was it due to you thinking what to do, or were there other things holding you back?
For ED Staff only	What happens to ensure effective handover of critical information to the anaesthetist when they arrive and how do you explain your decisions to them?
	If the anaesthetist comes without an ODP, what happens about offering them help with anaesthetic management?
	What happens if you disagree with the anaesthetist’s plan for airway management?
General	Thank you for exploring this with me. Do you have any final closing comments you would like to make? If you have any questions after the interview, then please don’t hesitate to contact me. Thank you for your time.
